# Comparative analysis of intraoperative thermal and optical imaging for identification of the human primary sensory cortex

**DOI:** 10.1117/1.JBO.30.1.016002

**Published:** 2025-01-16

**Authors:** Juliane Müller, Martin Oelschlägel, Stephan B. Sobottka, Matthias Kirsch, Gerald Steiner, Edmund Koch, Christian Schnabel

**Affiliations:** a TU Dresden, Carl Gustav Carus Faculty of Medicine, Anesthesiology and Intensive Care Medicine, Clinical Sensing and Monitoring, Dresden, Germany; b TU Dresden, Carl Gustav Carus Faculty of Medicine, Department of Neurosurgery, Dresden, Germany

**Keywords:** functional brain mapping, optical imaging, thermal imaging, neurosurgical imaging techniques

## Abstract

**Significance:**

The precise identification and preservation of functional brain areas during neurosurgery are crucial for optimizing surgical outcomes and minimizing postoperative deficits. Intraoperative imaging plays a vital role in this context, offering insights that guide surgeons in protecting critical cortical regions.

**Aim:**

We aim to evaluate and compare the efficacy of intraoperative thermal imaging (ITI) and intraoperative optical imaging (IOI) in detecting the primary somatosensory cortex, providing a detailed assessment of their potential integration into surgical practice.

**Approach:**

Data from nine patients undergoing tumor resection in the region of the somatosensory cortex were analyzed. Both IOI and ITI were employed simultaneously, with a specific focus on the areas identified as the primary somatosensory cortex (S1 region). The methodologies included a combination of imaging techniques during distinct phases of rest and stimulation, confirmed by electrophysiological monitoring of somatosensory evoked potentials to verify the functional areas identified by both imaging methods. The data were analyzed using a Fourier-based analytical framework to distinguish physiological signals from background noise.

**Results:**

Both ITI and IOI successfully generated reliable activity maps following median nerve stimulation. IOI showed greater consistency across various clinical scenarios, including those involving cortical tumors. Quantitative analysis revealed that IOI could more effectively differentiate genuine neuronal activity from artifacts compared with ITI, which was occasionally prone to false positives in the presence of cortical abnormalities.

**Conclusions:**

ITI and IOI produce comparable functional maps with moderate agreement in Cohen’s kappa values. Their distinct physiological mechanisms suggest complementary use in specific clinical scenarios, such as cortical tumors or impaired neurovascular coupling. IOI excels in spatial resolution and mapping reliability, whereas ITI provides additional insights into metabolic changes and tissue properties, especially in pathological areas. Combined, these modalities could enhance the understanding and analysis of functional and pathological processes in complex neurosurgical cases.

## 1 Introduction

Neurosurgical procedures require not only exceptional precision but also a comprehensive anatomical understanding to localize critical functional brain areas and ensure their preservation. One of these critical regions is the primary somatosensory cortex, essential for processing sensory stimuli from different parts of the body. Maintaining the functionality of this region during surgical interventions is important for preserving patients’ postoperative quality of life. There are various techniques available, such as functional magnetic resonance imaging, positron emission tomography, and diffusion-weighted imaging.^1^^,^^2^ Although these methods are highly valuable for risk evaluation and preoperative planning,^3^ they present a drawback: functional imaging data are collected before surgery and then mapped to the surgical site using neuronavigation systems. This process has the limitation that post-craniotomy; due to potential brain shifts, the precision of the image fusion is uncertain. Research has indicated that depending on the registration method used, discrepancies of up to 5 mm can occur.^4^^,^^5^ The development and implementation of highly precise intraoperative imaging techniques are therefore pivotal in allowing surgeons to identify and preserve functional areas in real time.

Intraoperative optical imaging (IOI) is a functional imaging technique that provides information about the optical properties of brain tissue by detecting changes in oxygenation and regional cerebral blood volume (rCBV).^6^^–^^8^ This technique leverages the different absorption characteristics of oxygenated and deoxygenated hemoglobin within the visible light spectrum to detect minimal variations in backscattered light caused by hemodynamic changes. IOI enables precise mapping of functional brain areas with high temporal and spatial resolution, which is particularly useful during complex neurosurgical procedures. It is especially effective in mapping somatosensory and motor areas,^9^^,^^10^ as well as for the visual cortex.^11^ Meyer et al.^12^ and Oelschlägel et al.^13^ demonstrated that IOI provides reliable and accurate results, for instance, in mapping the somatosensory cortex’s response to electrical stimulation.

In parallel, the use of intraoperative thermal imaging (ITI) has been explored as an alternative technique for functional brain mapping. This technique is based on detecting the brain’s surface temperature. Temperature changes triggered by neuronal activity or altered perfusion are captured and visualized by ITI in real time. Gorbach et al. demonstrated the feasibility of ITI for intraoperative brain mapping by detecting temperature changes associated with underlying regional cerebral blood flow (rCBF) and metabolic processes caused by increased neuronal activity.^14^^–^^16^ However, accurately detecting and interpreting these often very small temperature differences remain a challenge as they can be influenced by confounding factors such as ambient conditions and patient-specific physiological variations.

Compared with electrophysiological measurement methods, both IOI and ITI offer the advantage of combining spatial and temporal data acquisition in a single measurement. This is particularly useful during surgery as these imaging techniques allow for immediate visual feedback, enabling the surgeon to observe and identify functional brain areas in nearly real time. Electrophysiological measurements provide only a temporal representation of electrical activity at specific points, with no direct spatial visualization of the activated brain regions. IOI and ITI are thus offering a more comprehensive view of brain functionality during surgery.

In this article, we present a further optimized application of ITI that has the potential to detect functional areas with precision comparable with that of IOI. Furthermore, we describe a comprehensive patient study aimed at comparing both imaging techniques. The objectives of this study are to compare the spatial and temporal characteristics of functional activation in the primary sensory cortex as captured by ITI and IOI, analyze the signal amplitude and temporal dynamics of both imaging modalities during functional activation, and evaluate the roles of ITI and IOI in intraoperative functional mapping. By gaining a deeper understanding of the strengths and limitations of these modalities, this study seeks to determine how ITI and IOI can be most effectively utilized to enhance the safety and outcomes of neurosurgical interventions. Ultimately, the findings aim to contribute to the refinement of intraoperative imaging strategies and improve surgical outcomes for patients undergoing brain surgery.

## 2 Materials and Methods

### 2.1 Patients and Data Acquisition

In this study, we included nine patients undergoing neurosurgical tumor resections involving the primary somatosensory (S1) cortex, as listed in Table 1. Patients’ ages ranged from 32 to 81 years, with a median age of 69 years. None of the patients exhibited relevant sensory deficits prior to their neurosurgical intervention, ensuring that pre-existing neurological conditions did not influence the outcomes of the imaging assessments. All patients provided informed consent prior to their participation, and the study was approved by the institutional ethics committee of the Technische Universität Dresden in accordance with the Declaration of Helsinki.

**Table 1:** Characteristics of patients included in this study.

Case no.	Age	Sex	Pathology	Hemisphere
*P1*	72	*f*	Glioblastoma multiforme	*R*
*P2*	58	*m*	Metastasis (bronchial carcinoma)	*L*
*P3*	69	*m*	Glioblastoma multiforme	*L*
*P4*	72	*m*	Astrocytoma	*L*
*P5*	79	*m*	Metastasis (melanoma)	*R*
*P6*	70	*f*	Glioblastoma multiforme	*L*
*P7*	49	*f*	Metastasis (melanoma)	*L*
*P8*	81	*m*	Metastasis (melanoma)	*L*
*P9*	32	*f*	Meningeoma	*L*

f, female; m, male; R, right; L, left

All patients underwent preoperative magnetic resonance imaging (MRI) to obtain detailed anatomical data of the brain. ITI and IOI were performed simultaneously directly after the craniotomy. The hand area of each patient was stimulated via electrical stimulation of the contralateral median nerve at the wrist using a transcutaneous bipolar electrode and a standard neurostimulation device (Bravo/Endeavor; Viasys, Nicolet Biomedical, Madison, Wisconsin, United States). According to clinical standards, a stimulation current of I=20  mA and an electrical stimulation frequency of f=5.1  Hz were used.^17^ The stimulation protocol comprised a 9-min scheme with alternating 30-s rest and 30-s stimulation trials. Verification of the S1 cortex location within the areas captured by ITI and IOI was further confirmed through electrophysiological measurement of somatosensory evoked potentials (phase reversal). All information including trepanation and location of the S1 region and, if applicable, the motor cortex, when trepanned, was displayed on a 3D model of the brain reconstructed from the preoperative MRI.

### 2.2 Intraoperative Optical Imaging

IOI is a high-resolution imaging technology that detects minimal variations in cortical optical reflectance, primarily triggered by neuronal activation and the resultant functional hyperemia. These variations are observed as changes in reflectance, captured in this study with a monochrome charged-coupled device (CCD) camera, a band-pass interference filter, and a microscope-integrated xenon light source, as listed in Table 2. Light wavelength filtering was performed within the optical path at λ=568±5  nm, targeting blood volume changes. Image datasets were acquired at 5 to 15 frames per second.

**Table 2:** Specifications of the imaging hardware setup.

ITI	
Camera	VarioCAM HD head (Infratec GmbH, Dresden, Germany)
Detector	Uncooled microbolometer focal plane array
Spectral measurement range	(7.5 … 14) μm
Thermographic temperature range	(–40 … 1200)°C with special calibration for medical applications: (20 … 40)°C
Temperature resolution at 30 °C	Better than 30 mK (low noise detector)
Absolute measurement accuracy	±1 K
Spatial resolution	640 × 480 pixels, object distance of 30 cm: 125 μm/pixel
Frame rate	60 Hz
Microscope	Zeiss OPMI Pico
**IOI**	
Camera	AxioCam MRm (Carl Zeiss MicroImaging, Jena, Germany)
Filtering	Bandpass interference filter, central wavelength $\lambda_{c}=568\;\mathrm{nm}$, FWHM = 10 nm (Edmund Optics, Barrington, New Jersey, United States)
Exposure time	50 ms
Spatial resolution	1388 × 1040 pixels (694 × 520 pixels with 2 × 2 binning)
Frame rate	5 to 15 Hz
Digitization	12 bit
Data transfer via	IEEE 1394 FireWire
Microscope	Zeiss OPMI Pico/Zeiss OPMI Pentero
Illumination (microscope integrated)	Xenon 180 W/Xenon 300 W

The chosen wavelength (568 nm) corresponds to an isosbestic point of hemoglobin absorption, where both oxy- and deoxygenated hemoglobin have nearly identical absorption coefficients. This ensures that the detected signal is equally sensitive to both components, providing a measure of changes in CBV. The penetration depth of the 568-nm wavelength is less than 1 mm, which is ideal for capturing activity in the superficial cortical layers. Previous studies have demonstrated that this wavelength reliably generates functional activation maps of the primary sensory cortex.^12^^,^^13^

### 2.3 Intraoperative Thermal Imaging

ITI captures temperature variations on the brain surface, which are indicative of underlying neuronal activities and correlated with rCBF and metabolic changes. Therefore, ITI utilizes high-resolution infrared cameras. This study was conducted using the uncooled infrared (IR) camera system VarioCAM HD head (Infratec GmbH, Dresden, Germany). This IR system operates in the long-wave infrared range, specifically from 7.5 to 14  μm. It also requires less apparatus than cooled IR systems, making it better integrated into the operating room and more cost-effective. The lens used projects the object onto a microbolometer focal plane array with a dimension of 640×480  pixels, providing a resolution of 125  μm/pixel at an object distance of 30 cm and a temperature resolution of 30 mK (at 30°C). The specifications of the IR system are also summarized in Table 2.

### 2.4 Data Analysis

The data analysis for IOI was conducted using Fourier analysis, as described in previous publications.^10^^,^^12^^,^^13^ This approach detects periodic reflectance changes in response to alternating stimulation and rest trials. The resultant frequency components generate functional activation maps that visually represent cortical activity.

The analysis of ITI data followed the same approach, employing a Fourier-based analytical framework with adapted preprocessing. This method identifies periodic temperature changes in brain regions by transforming the temperature signal into the frequency domain to identify periodic signal components. These components indicate neuronal activity in the studied brain regions, allowing the generation of activity maps that visually represent the location and extent of cortical activation.

Several critical preprocessing steps are necessary before applying the discrete Fourier transform (DFT), especially on ITI data. First, non-uniformity correction (NUC) artifacts, specific for automatic recalibration of IR cameras as described in Ref. 18, are addressed. These artifacts appear as abrupt changes in the temperature profile and are adjusted by setting the derivative of the temperature data at these points to 0, effectively smoothing out abrupt transitions. Next, the original sampling rate of 60 Hz is reduced to 10 Hz to manage data volume and computational demand while retaining sufficient detail to analyze changes in rCBF related to neural activation. The sampling rate reduction is done after NUC correction to avoid masking the artifacts. Pixels representing the cortical brain surface are initially identified and segmented using automatic edge detection techniques. These regions of interest (ROIs) correspond to the brain surface exposed by the trepanation and are critical for identifying the relevant functional areas, such as the S1 cortex. In cases where the automatic methods did not produce satisfactory results—particularly in ITI due to artifacts of fluids at the edges of the trepanation—manual ROI selection was employed.

This manual selection was performed in conjunction with the registered IOI images, ensuring precise identification of the functional areas associated with the median nerve stimulation, particularly in the hand area of S1. By aligning the ROI placement with the registered IOI images, we were able to ensure consistent and accurate comparisons between IOI and ITI.

Slow temperature drifts due to environmental factors are compensated for using cubic polynomials to model these changes, subtracting the fitted polynomial from the original signal to eliminate low-frequency temperature fluctuations.

After preprocessing, the data undergo DFT using the fast Fourier transform (FFT) algorithm. After performing the FFT, the resulting spectrum is used to calculate the power spectral density (PSD), determined by the square of the magnitude of the Fourier transform.^19^ For stimulation analysis, the power of the stimulation signal ${\mathrm{PSD}}_{S}$ at the stimulation frequency $f_{S}=0.0167\;\mathrm{Hz}$ (corresponding to 30-s rest and 30-s stimulation) is calculated pixel by pixel. This frequency is in the range relevant to cerebral autoregulation. According to Ref. 20, these frequencies are categorized into two groups: very low frequencies (VLF, 0<f≤0.04  Hz) and low frequencies (LF, 0.04<f≤0.15  Hz). To minimize the influence of these low-frequency signals on the resulting activity maps, the power of the stimulation signal ${\mathrm{PSD}}_{S}$ is normalized to the sum of the powers of the autoregulatory processes within the same frequency band, ${\mathrm{PSD}}_{\mathrm{VLF}}$ resulting in ${\mathrm{PSD}}_{\text{Norm}}$. This normalization is an essential step to ensure that the activity maps highlight only those regions with amplitudes that clearly exceed the contributions of autoregulation. In this way, specific regions of brain activity that respond to stimulation can be effectively distinguished from the background processes of cerebral autoregulation.

The validation of ITI and IOI methods involves correlating the activity maps with anatomical references from preoperative MRI scans and functional benchmarks derived from intraoperative electrophysiological measurements. These electrophysiological data, such as evoked potentials, are used both to validate the accuracy of the detected neuronal activity and to assist in the registration and alignment of the intraoperative imaging modalities (ITI and IOI). This ensures that the functional maps generated by both methods are reliable and contribute effectively to the identification of brain activity during surgery.

To ensure accurate comparison and visualization of the IOI and ITI data, we performed manual image co-registration. This was achieved by selecting X–Y control points in the averaged IOI and ITI images and applying an affine transformation to align the datasets. In addition, the IOI image sequence was corrected for brain surface movement using an elastic image registration based on the Demons algorithm prior to co-registration. This step ensured the alignment of IOI images over time, accounting for intraoperative brain shifts or motion artifacts.

For future clinical applications, co-registration could be automated. In a fixed imaging setup, this would involve a pre-calibrated transformation matrix to overlay images in real time, provided that the geometric alignment between the visual and thermal camera systems remains unchanged.

For visualization, the activity map of both modalities was filtered with a 3×3 Gaussian kernel to reduce noise, then color-coded, and projected onto a representative ITI or IOI image using an adjustable threshold. For quantitative analysis, the signal strength within the activated regions of the two imaging modalities, ITI and IOI, was evaluated and compared. The activated regions were defined as the areas in the activity map where the value exceeded a dynamic threshold. This dynamic threshold t was calculated as the sum of the mean intensity $\overset{¯}{I}$ of all activity map pixels within the trepanned region and their corresponding standard deviation

$$t=\overset{¯}{I}+\mathrm{std}(I).$$

**Fig. 1:**
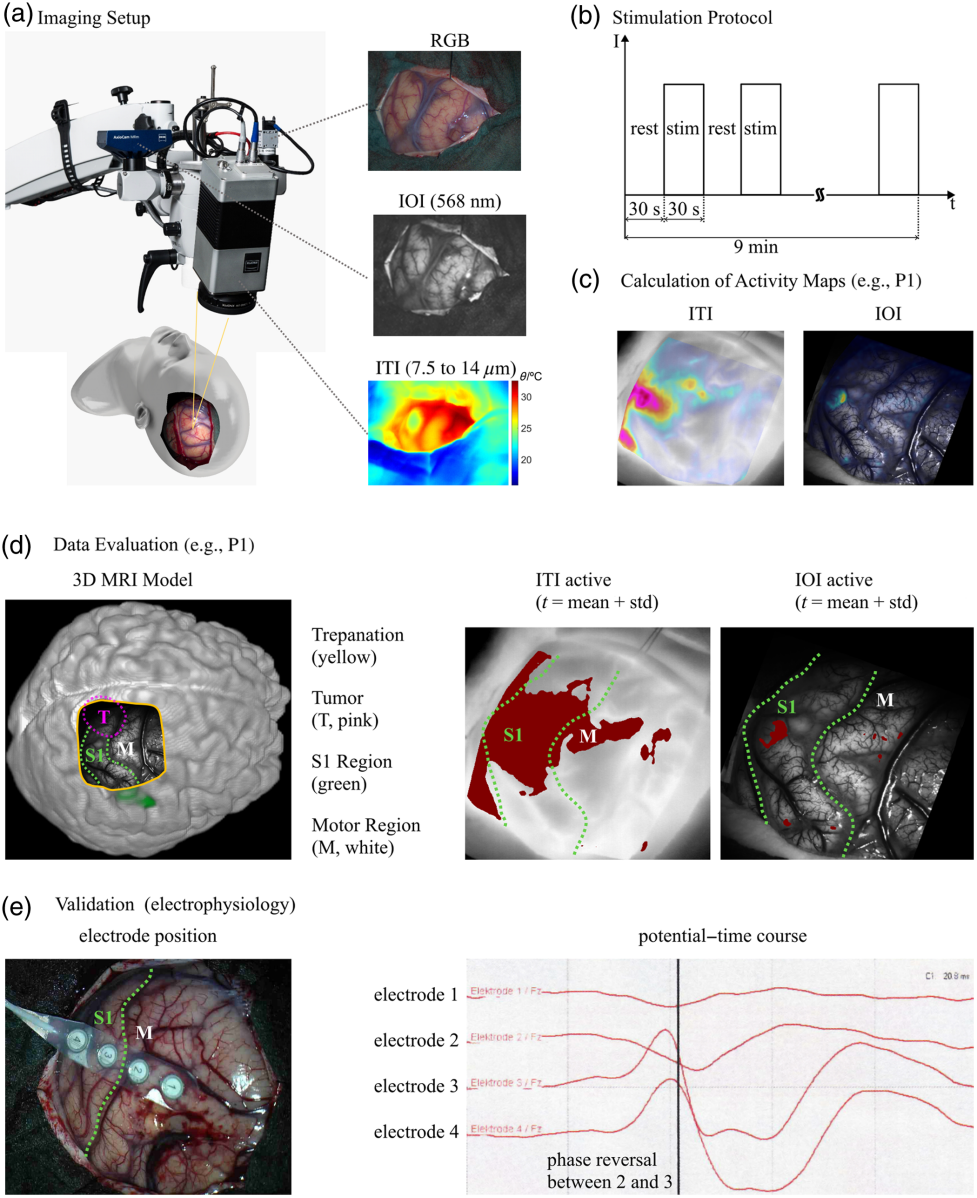
Overview of materials and method. In panel (a), the used imaging setup consisting of IOI at 568 nm, ITI, and an RGB camera is shown. Panel (b) illustrates the stimulation protocol of electrical stimulation of the median nerve with 30-s rest and 30-s stimulation (stim) phases for a total of 9 min. After data analysis, the activity maps presented in panel (c) are obtained and visualized by fusing them with corresponding thermal or visual images, as illustrated here with an example from P1. For the comparison of IOI and ITI in this paper, different regions are defined, which are explained in more detail in panel (d). This results in masks for the different regions: trepanation, S1 region, motor region, tumor, area defined as the active region in the ITI as well as area recognized as active in the IOI (for both all pixel values above the dynamic threshold). An example has been included in panel (e), showing the intraoperative electrode positions during the measurements of evoked potentials, to further illustrate how electrophysiological data are integrated into the validation process.

An overview of the study design is given in Fig. 1.

Cohen’s kappa was employed to evaluate the agreement between ITI and IOI in identifying functional activation areas. This statistical measure assesses the level of agreement between the two modalities by accounting for the possibility of random agreement. The analysis was performed pixel-wise, classifying each pixel as either “activated” or “non-activated” for the S1 and non-S1 regions, as well as the entire trepanation area. This approach ensures a detailed comparison of the activation areas detected by both modalities, providing a robust measure of their agreement.

## 3 Results and Discussion

### 3.1 General Characteristics of Functional Activation

**Fig. 2:**
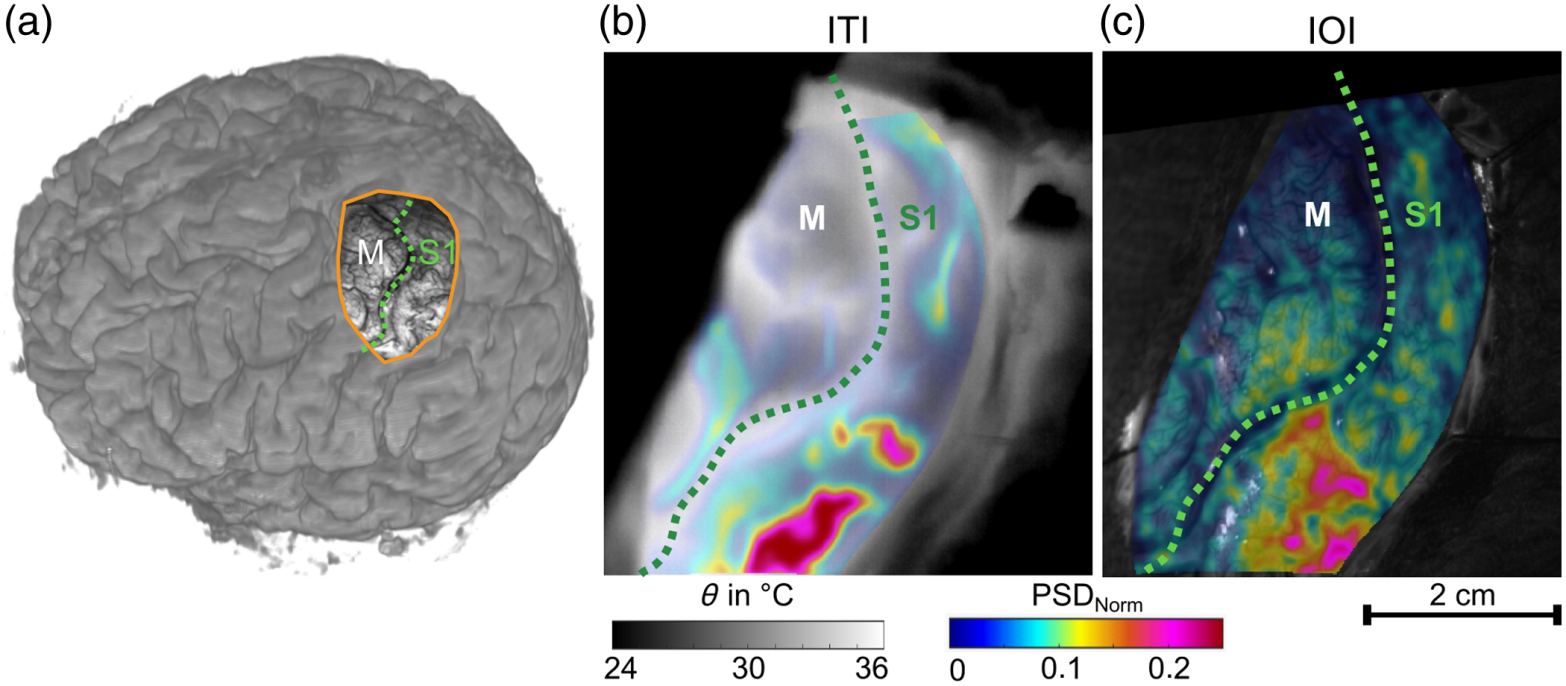
Comparison of the calculated ITI and IOI activity maps following median nerve stimulation for patient P8. In panel (a), an overview of the anatomical location of the trepanation and the eloquent areas (S1 and M) is given, illustrated by the preoperative 3D MRI reconstruction. Panel (b) presents the ITI activity map, and panel (c) displays the IOI activity map. Both maps show increased PSD_Norm values and, thus, significant activation in the primary somatosensory cortex.

Both ITI and IOI were able to generate reliable activity maps with activation in the hand area of the S1 region in all nine patients following median nerve stimulation. The results from patient P8 in Fig. 2 are representative of the majority of patients in this study. The activity maps of both modalities show a delineated activation in the S1 region validated by phase reversal. The activity in the surrounding areas is lower and more diffusely arranged.

As detailed in Table 2, the difference in spatial resolution contributes to the variation in the size of the activated areas. Due to the coarser spatial resolution of ITI, the activated areas appear more compact as broader regions of cortical activity are captured in fewer pixels, resulting in a concentrated visual representation. The finer spatial resolution of IOI allows for a more detailed depiction of the activated areas, leading to a broader and more distributed signal.

In addition, the noise level in ITI is slightly lower, and the signal amplitude of ${\mathrm{PSD}}_{\text{Norm}}$ is higher, which is likely due to the larger pixel size capturing a more aggregated signal over a larger area. The difference in signal origin between ITI and IOI may also play a role as ITI detects temperature changes related to neuronal activity, whereas IOI captures hemodynamic changes. These factors, including the resolution and signal type, likely explain the observed differences in the size and shape of the activation areas between ITI and IOI.

Although both IOI and ITI provide valuable information about functional brain activity, it is important to note that the S1 cortex may not be fully highlighted in the activity maps as the activation is dependent on the specific functional stimulation used during surgery. In our case, median nerve stimulation was employed, which activates only the corresponding area of the S1 region. This explains why only a part of the S1 area is visible in the activity maps in Figs. 1(d), 2(b), and 2(c).

Despite this partial activation, both IOI and ITI contribute notably to preserving the functionality of the S1 region. Traditionally, the intraoperative localization of the S1 cortex relies on electrophysiological measurements, such as identifying phase reversals between the motor and sensory cortices (N20/P20 potentials). However, this method requires multiple realignments and provides only point-specific information. IOI and ITI, on the other hand, allow the surgeon to visualize the spatial distribution of functional areas with a single measurement, providing visual feedback that can help preserve both the functionality and structural integrity of the S1 region. This capability is particularly beneficial for identifying and protecting critical areas during surgery, even if only part of the region is directly stimulated.

**Fig. 3:**
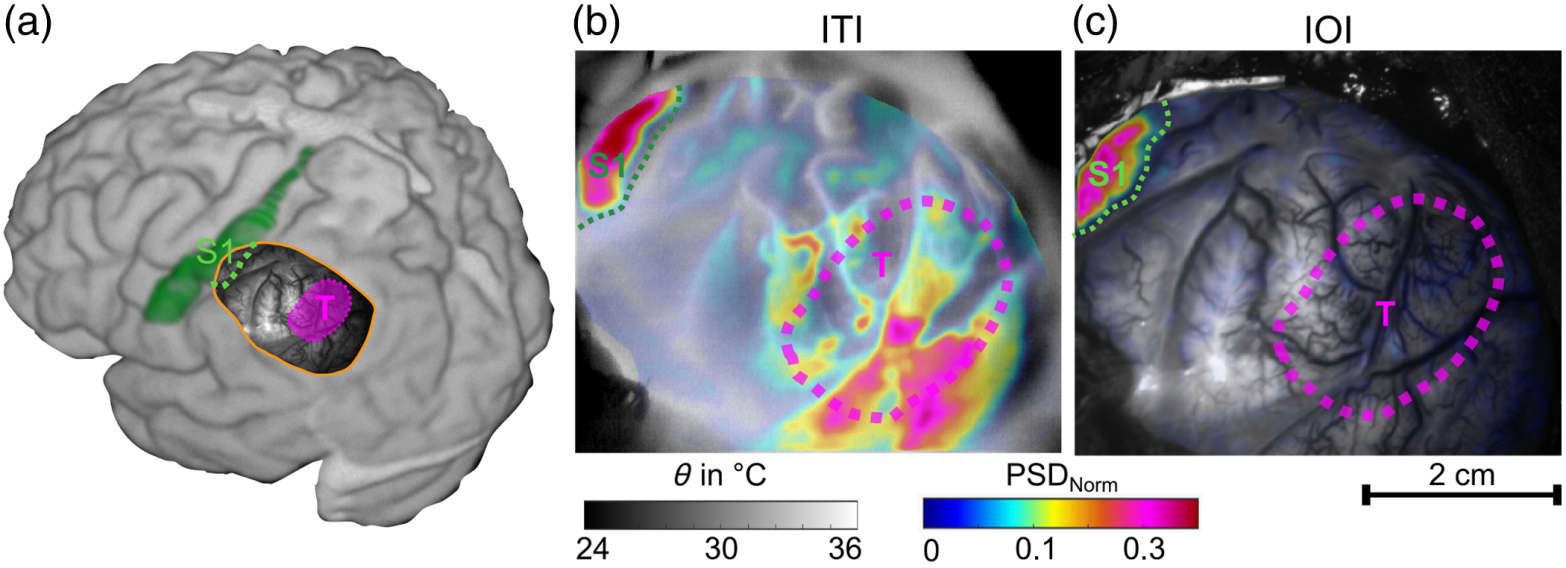
Results of the detection of the S1 region in patient P3. The activation matches well with the exposed portion of the S1 area, which is relatively small compared with the entire trepanation. However, this patient also has cortical tumor components (T) in the trepanation area, outlined in pink. These affect the ITI activity determination in this region, leading to false-positive results. The results of IOI do not show an influence of such effects. For better detection and to avoid these effects in the ITI, the threshold could be adjusted upward accordingly. Panel (a) shows the preoperative 3D MRI reconstruction with the anatomical location of the trepanation, the cortical tumor portion (T) and the S1 area. Panels (b) and (c) illustrate the ITI activity map and the IOI activity map, respectively.

In patients with cortical tumor components, such as P3 or P9, deviations are observed compared with the other patients. The cortical tumor components have a direct impact on the ITI measurements. Figure 3 illustrates the case of P3, where the tumor is located outside the S1 region. Although both ITI and IOI are able to accurately identify the exposed area of the sensory cortex, the ITI shows an apparent activation in the tumor region as well. This leads to false-positive results in the ITI activity map, which do not occur in the IOI. Tumor tissue also exhibits increased metabolism, which is likely being detected here. That this, signal behaves differently than the activation originating from the electrical stimulation of the median nerve, which becomes clearer if looking at the temperature dynamics described later and displayed in Fig. 6.

**Fig. 4:**
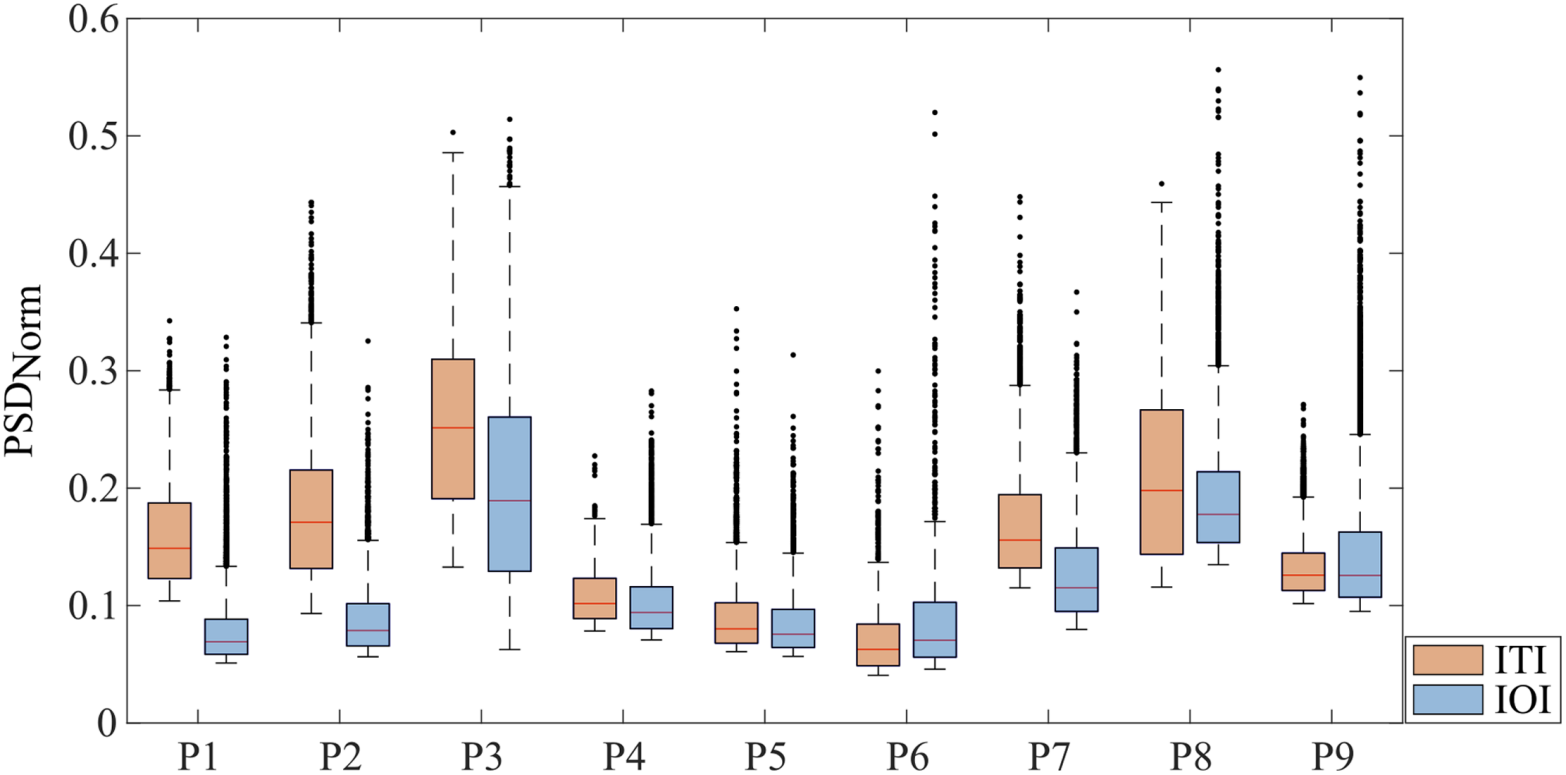
Representation of signal intensities in the regions of the S1 area defined as active by the respective imaging methods. The normalized power spectral density (PSD_Norm) generally shows similar values, with the exception of P1 and P2, where the PSD_Norm values for ITI (orange) are slightly higher than those for IOI (blue). In some cases, the median for ITI is above that of IOI, indicating higher PSD_Norm values. The variability is also somewhat higher in some ITI data, showing a broader distribution of activity values in the S1 region.

The exact distribution of activity values above the dynamic threshold for both IOI and ITI for all patients is shown in Fig. 4. In many cases, the medians of the ${\mathrm{PSD}}_{\text{Norm}}$ values for ITI and IOI were in a similar range. However, exceptions were found in patients P1 and P2, where the medians for ITI were notably higher than those for IOI. In addition, ITI showed a broader distribution of activity values, indicating greater variability in signal intensities within the studied sample. This may be attributed to different tissue response patterns or technical aspects of the ITI method itself.

It is important to note that the dynamic threshold used in this study is dependent on the ratio between the size of the trepanned region and the size of the exposed S1 cortex. Although this dependency makes the dynamic threshold unsuitable for quantitative comparisons among different patients, it is well-suited for intra-patient comparisons of IOI and ITI. A static threshold, by contrast, would be problematic due to the normalization of the PSD at the stimulation frequency to the power in the VLF band. Variations in VLF oscillations within a single patient could lead to inconsistent results if a static threshold were applied, making the dynamic threshold the more appropriate choice for this study.

To assess the accuracy and reliability of both methods, the proportion of activated pixels relative to the total number of pixels corresponding to, or not corresponding to, the anatomical somatosensory cortex was examined.

**Fig. 5:**
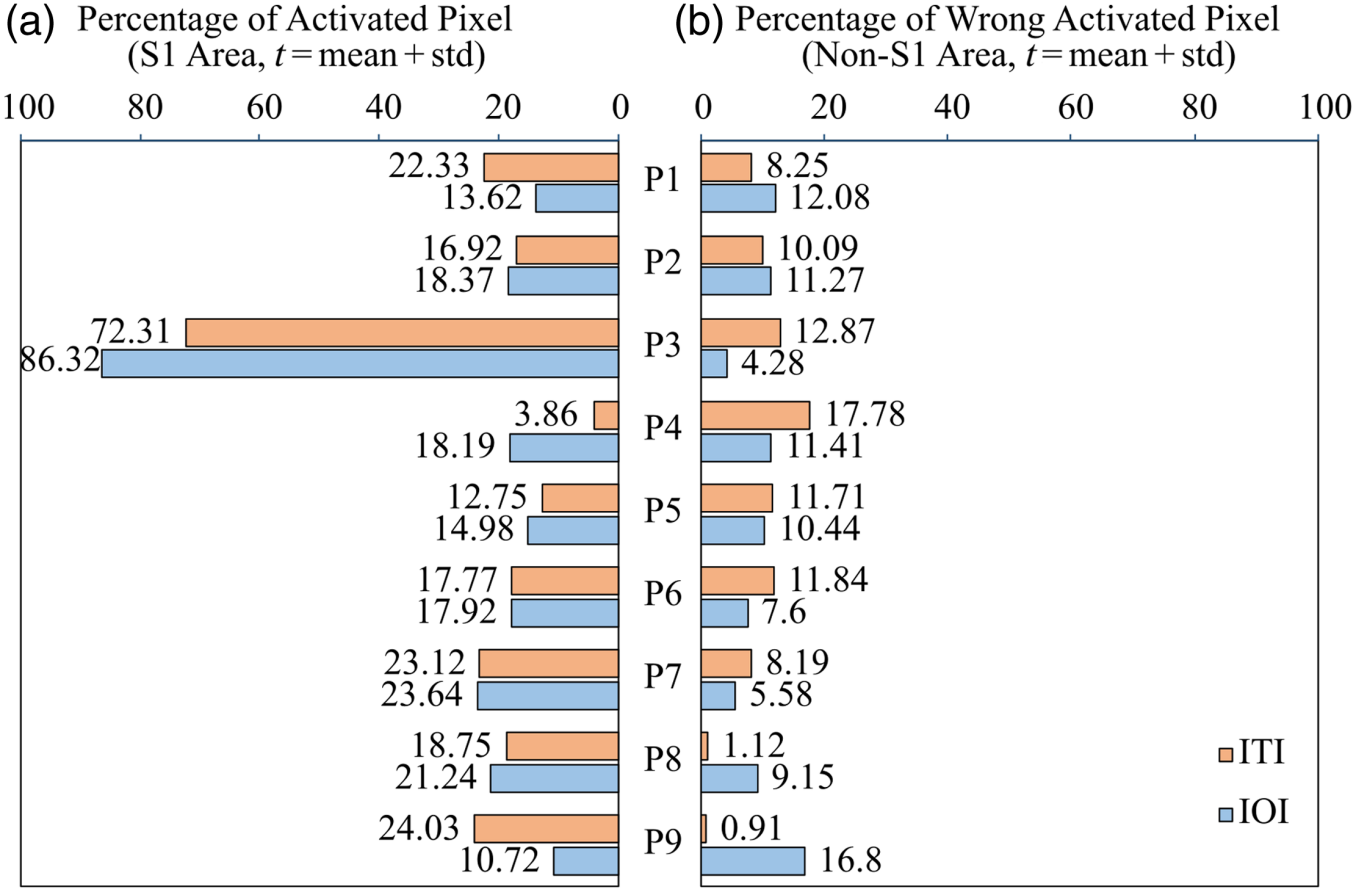
Percentage of activated pixels in relation to the S1 area between ITI (orange) and IOI (blue). (a) True-positive cases where the activated pixels were identified within the S1 area. (b) False-positive cases where activation was detected outside the S1 area.

Figure 5 shows the true-positive pixels on the left (activations correctly localized within the S1 area) and the false-positive pixels on the right (activations outside the S1 area). Both imaging methods demonstrate similar accuracy and reliability. With the exception of one patient (ITI—P4, IOI—P9), the proportion of activated pixels in the S1 region predominates. Despite occasional deviations, where one method shows slightly higher accuracy, the overall accuracy ratio in the studied sample remains largely balanced. A special case in this study was patient P3, where the S1 region was only partially exposed in a relatively small area. This resulted in a relatively high proportion of activated pixels within the S1 region. The generally low proportions of around 10% to 25% of activated pixels in relation to the anatomical somatosensory cortex can be explained by the fact that the postcentral gyrus is anatomically considered the S1 area. However, during stimulation, only the region of the median nerve, a part of the S1 area, was stimulated. In addition, the S1 area was not fully trepanned; instead, the extent of the trepanation was adapted individually to minimize the burden on the patient. The resulting proportion corresponds to the average proportion of the S1 area recognized as active by the imaging methods.

The percentage of incorrectly activated pixels, as shown in Fig. 5, is calculated relative to the exposed S1 and non-S1 areas. A false-positive rate of around 10% in the non-S1 region does not necessarily hinder the clear identification of the S1 area in the activity maps. This percentage does not specify whether the false-positive pixels are isolated, scattered, or form a larger connected area, which may affect their clinical relevance. Even with some false positives present, the primary sensory cortex (S1) remains visually identifiable, enabling the surgeon to differentiate it from surrounding areas and preserve its functionality.

In addition, the proportion of activated pixels is influenced by the extent of the exposed S1 area during surgery. If a large part of the S1 cortex is exposed, the activated area representing the median nerve will naturally constitute a smaller percentage of the total S1 region. In cases where only the median nerve area is exposed, this proportion will be higher. These differences in the percentage of activated pixels are primarily intended to compare the relative performance of IOI and ITI within the same patient, rather than to assess absolute clinical accuracy across different patients.

Despite these observed inaccuracies, both IOI and ITI provide valuable intraoperative visual feedback that can assist the surgeon in identifying and preserving crucial functional areas. This real-time visual feedback is the primary utility of these methods, ensuring the integrity of essential brain regions during surgery.

The broader distribution of activity values in ITI could indicate a greater diversity in the detected signals, highlighting ITI’s ability to capture a wide range of physiological states. This variability could serve as a valuable source of information to better understand the complexity of brain functions and investigate potential influences of external factors or pathological changes on brain activity in subsequent studies.

### 3.2 Analysis of Signal Dynamics

**Fig. 6:**
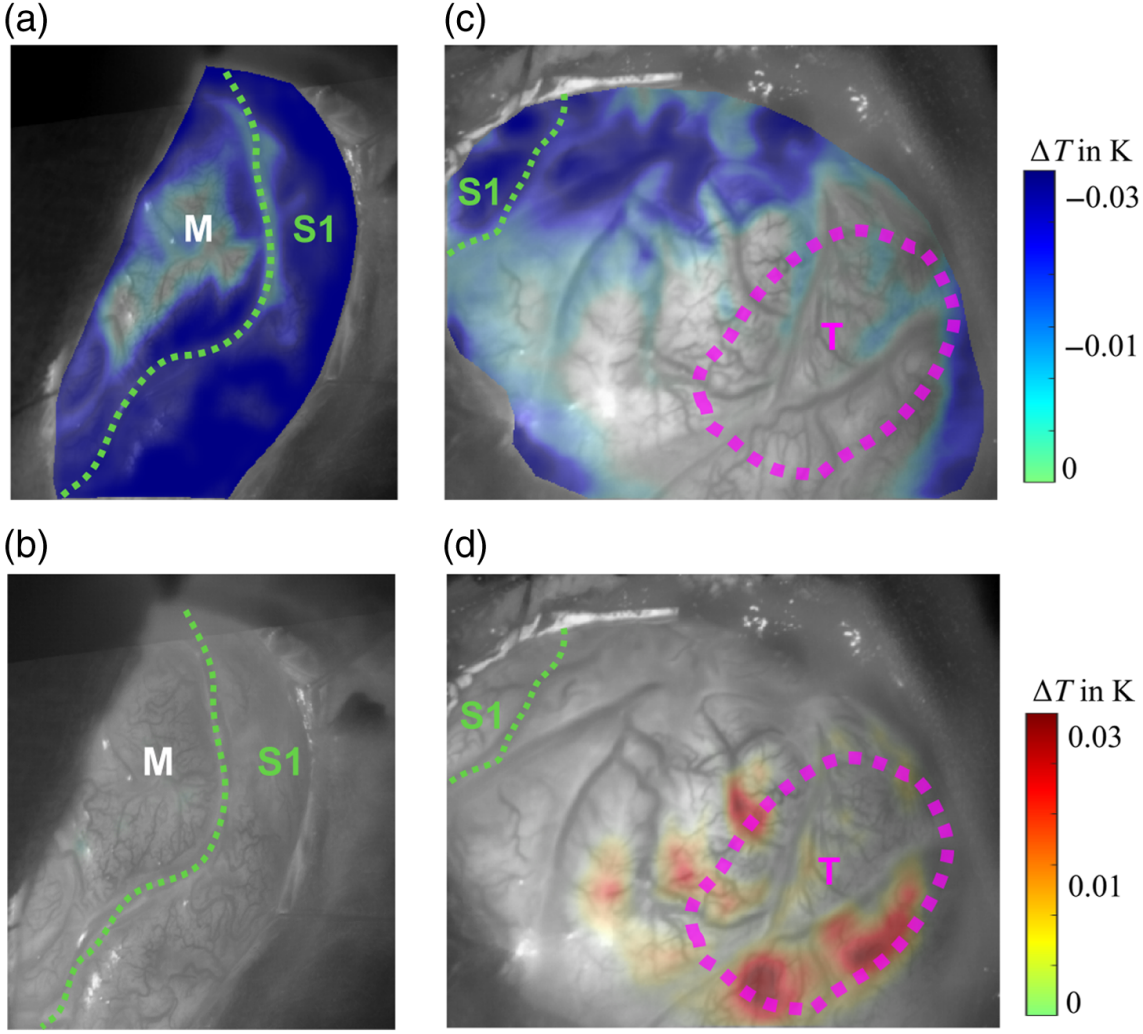
Temperature dynamics between rest and stimulation phases in patient P8 (a), (b) and P3 (c), (d). Each panel shows the difference in temperature between the baseline temperature of the time series and the mean temperature in rest and stimulation phases for the selected patients. In panels (a) and (c), the images show areas where the temperature was lower during the stimulation phase compared with the rest phase. Each colored pixel in these images indicates a cooling of that region during stimulation. By contrast, panels (b) and (d) highlight areas where the temperature was higher during the stimulation phase. For P8 in panel (b), no regions show a temperature increase during stimulation, meaning that the temperature was consistently lower across the trepanation area during stimulation, which was typical for most patients in this study. In panel (d), for patient P3, certain areas in the tumor region (T) showed an increase in temperature during stimulation, indicating a different behavior in the pathologically altered tissue.

An initial step in investigating these hypotheses is the analysis of the mean temperature differences between rest and stimulation phases across different regions of the cerebral cortex to gain more detailed insights into temperature dynamics. The investigation of temperature dynamics during rest and stimulation phases in patients P8 and P3, shown in Fig. 6, yields important insights. The analysis shows that in stimulated cortex regions, the temperature is lower during the stimulation phases than during the rest phases. Notably, in patient P8, no areas were identified where the temperature was higher during the stimulation phases than in the rest phases. This pattern, which shows a general cooling effect in the affected brain areas during stimulation, is characteristic of the majority of patients in this study.

**Fig. 7:**
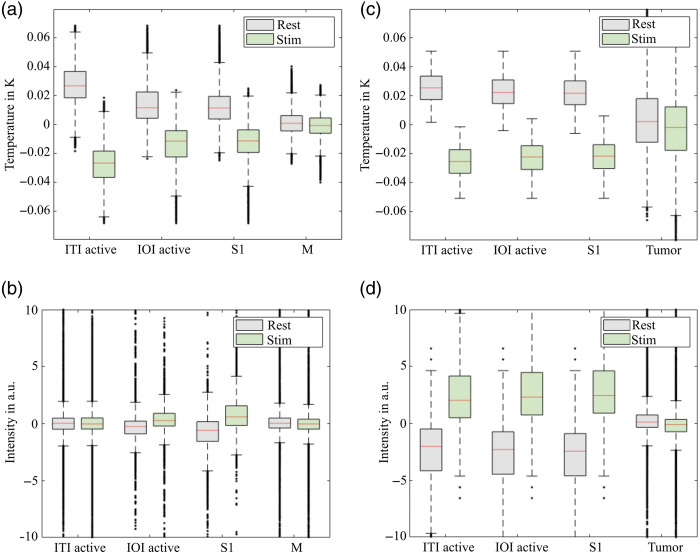
Relative temperature (recorded with ITI) and intensity (recorded with IOI) in relation to the mean value of the time series (9 min, zero value in the y-axis) for P8 (a), (b) and P3 (c), (d) for different regions (defined in Fig. 1). These diagrams clearly show that the temperature in the S1 area is generally higher during the rest phase than during the stimulation phase. This is especially evident in the regions identified as active in ITI (ITI active) but also in those activated in IOI (IOI active), and a trend is also noticeable in the entire S1 area. By contrast, the temperature dynamics in the motor cortex (M), which was not stimulated, showed no clear differences between rest and stimulation. The tumor area (T) in P3 exhibits opposite behavior between the rest and stimulation phases, which appears more pronounced in the temperature signal in panel (c) than in the intensity signal of the IOI in panel (d). The temperature differences between the rest and stimulation phases are also reflected in the intensities (b), (d). The intensity signal increases during stimulation, indicating an increase in the reflectance of the region and thus a reduction in rCBV during stimulation.

Furthermore, a detailed examination of temperature changes in the S1 area in Fig. 7, subdivided into regions activated by IOI and ITI, provides deeper insights into the brain’s physiological responses to stimulation. The areas where ITI detects active pixels are the regions with the greatest temperature difference. These findings underscore the specificity of temperature changes in the S1 area in response to stimulation. Unlike the motor cortex, which does not show such a response, the targeted and specific response of the S1 area to neurological stimulation is highlighted.

In analyzing the temperature dynamics during rest and stimulation phases in patient P3, interesting observations were made, especially when comparing the S1 region and the tumor region. In the S1 region, a typical temperature decrease was observed during stimulation, indicating activation of this region. By contrast, the pathologically altered tissue in the tumor region showed different behavior. Here, areas could be identified that exhibited a temperature increase during stimulation. Moreover, the tumor tissue caused a broad distribution of temperature values in both phases, suggesting impaired neurovascular coupling. This impairment manifested as a false-positive activation in ITI but with different temperature behavior compared to the S1 region. Additional data regarding these temperature dynamics and further examples can be found in the Supplementary Material.

These observations underscore the importance of a differentiated examination of temperature dynamics in various tissue types and the need to consider these factors when interpreting activity maps. A decrease in temperature during stimulation may indicate a reduction in rCBF in the stimulated areas. This may seem paradoxical as an increase in blood flow is usually expected during neural activation. Nevertheless, it has already been described by Oelschlägel et al. that sustained electrical stimulation leads to a decrease in rCBV and thus an increase in IOI intensity (as seen in Fig. 7). By contrast, sustained tactile and visual stimulation resulted in an increase in rCBV in the correspondingly activated areas. In awake patients performing language tasks, areas with both increased and decreased rCBV were observed. These findings suggest that decreases in rCBV in the primary sensory cortex are associated with the processing of nociceptive stimuli and that the type and paradigm of stimulation notably influence the hemodynamic response.^13^

This observation indicates a potential reduction in rCBF during electrical stimulation that cannot be directly measured with IOI. ITI provides additional thermodynamic information, which highlights metabolic changes and may offer complementary insights into pathological conditions. The relationship among neural activity, blood flow, blood volume, and temperature is complex and not always directly proportional. Different types of stimulation and varying physiological or pathological states of brain tissue (e.g., tumor presence) can alter neurovascular coupling, leading to distinct patterns that reflect the unique strengths of ITI and IOI.

### 3.3 Spatial Correlation of Functional Activation in ITI and IOI

The agreement between the activity regions identified by ITI and IOI was assessed using Cohen’s kappa, a statistical measure that accounts for random agreement. For the analysis, binary masks of the ITI and IOI activity maps were created based on the activity regions defined by dynamic thresholds. These masks were applied to the entire trepanation region, the S1 region, and the non-S1 regions.

Cohen’s kappa values indicate that the agreement between ITI and IOI is generally higher in non-S1 regions, where kappa values range from 0.68 to 0.88, reflecting substantial to almost perfect agreement. This is likely because non-S1 regions are less functionally complex, with fewer areas of activation, leading to clearer identification of “non-activated” regions by both modalities.

By contrast, the S1 region shows more variability in agreement, with kappa values ranging from 0.42 to 0.69, indicating moderate to substantial agreement. The lower kappa value for patient P7 (0.42) highlights the variability in this region possibly due to differences in noise levels or signal origins between the two methods, as well as the more intricate functional activity in the S1 region.

These differences can be attributed to the distinct technical characteristics of ITI and IOI. ITI tends to detect more compact activation regions due to its lower spatial resolution and lower noise levels, whereas IOI, with its higher spatial resolution, often shows larger or more diffuse activation areas. In addition, the dynamic thresholding method used in both modalities can lead to variations in the size of the detected activation regions.

Table 3 summarizes Cohen’s kappa results for the S1 region, non-S1 regions, and the entire trepanation area.

**Table 3:** Results of Cohen’s kappa analysis.

Case no.	S1 region	Non-S1 region	Entire trepanation
*P1*	0.65	0.76	0.72
*P2*	0.63	0.76	0.72
*P3*	0.66	0.83	0.81
*P4*	0.69	0.69	0.69
*P5*	0.68	0.75	0.71
*P6*	0.64	0.79	0.77
*P7*	0.42	0.85	0.70
*P8*	0.61	0.88	0.77
*P9*	0.60	0.75	0.64

Overall, these findings suggest that ITI and IOI provide comparable functional maps in typical scenarios, particularly in non-S1 regions where functional activity is less complex. However, the variability in S1 regions indicates that each modality may capture different aspects of brain activity due to their distinct signal origins. These results underscore the need for further studies to explore the specific contexts in which ITI and IOI may provide complementary insights.

## 4 Conclusion

This study has successfully demonstrated that ITI and IOI yield comparable results in detecting the primary somatosensory cortex during neurosurgical interventions. Both imaging modalities have shown their capacity to generate reliable activity maps, with IOI providing more consistent results across different clinical scenarios, including cases complicated by the presence of cortical tumors.

Furthermore, the development of the Fourier-based analytical framework employed for processing ITI data made the robust application of ITI for functional characterization of the cerebral cortex possible in the first place and has proven particularly useful in isolating physiological signals from background noise, thereby enabling precise activity mapping.

Challenges remain in the application of ITI, particularly in its sensitivity to external factors and its interpretation in the context of cortical pathology. Future research should focus on refining ITI techniques to better differentiate between pathological and normal physiological responses in the brain. In addition, expanding the use of these imaging modalities to other sensory and functional areas of the brain could provide broader insights into cortical function and its implications for neurosurgical practice.

The results of this study showed that both ITI and IOI can accurately identify activated regions in the S1 area following median nerve stimulation. IOI provided higher spatial resolution, enabling a clearer delineation of activation regions, whereas ITI revealed a broader distribution of activity values, reflecting its capacity to capture a wide range of physiological states. The delayed and more prolonged response observed in ITI compared with IOI aligns with known hemodynamic-metabolic coupling.

These findings suggest that although ITI and IOI provide largely comparable functional maps in typical scenarios, their distinct signal origins offer potential complementary insights in specific contexts, such as cases involving cortical pathology. This underscores the value of a multimodal imaging approach in neurosurgery, leveraging the strengths of both ITI and IOI to enhance the accuracy of intraoperative functional mapping. However, the interpretation of imaging results still requires subjective decisions by the surgeon due to the variability of the data, as demonstrated throughout this study.

## Supplementary Material



## Data Availability

As these datasets contain patient-related information, they will be kept confidential to protect patient privacy. Access to anonymized data may be available from the corresponding author upon reasonable request and is subject to an approval process that includes an ethics review and a signed data access agreement.
