# Supplementary material for: Comparative analysis of intraoperative thermal and optical imaging for identification of the human primary sensory cortex
Source: J Biomed Opt. 2025 Jan 16;30(1):016002. doi: 10.1117/1.JBO.30.1.016002 (PMC11737595; doi:10.1117/1.JBO.30.1.016002)
Supplement: Supplementary file 1 [file JBO_030_016002_SD001.docx]

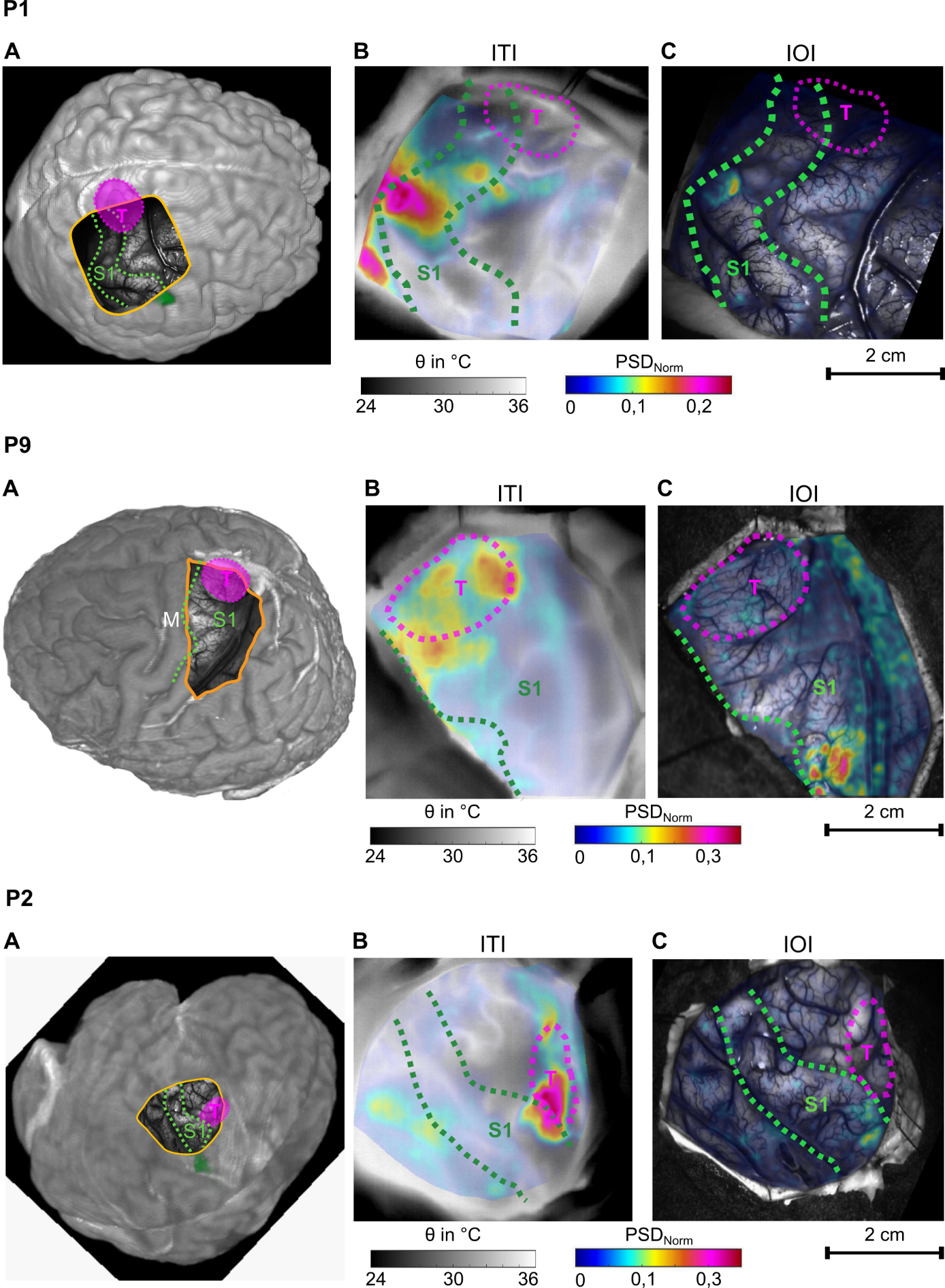


**Fig. Suppl. 1** Comparison of the detection of the S1 region in three patients with cortical tumors located at varying positions relative to the S1 region. In A, an overview of the anatomical location of the trepanation, the S1 region, and the tumor (outlined in pink) is shown, illustrated on the preoperative 3D MRI reconstruction. Image B presents the ITI activity map, highlighting the activation regions, including areas affected by the tumor. Image C displays the IOI activity map, showing activation limited to the S1 region without significant influence from the tumor. The results demonstrate that the tumor's proximity to the S1 region affects ITI activity determination, leading to false-positive results, while IOI is less affected by such artifacts.


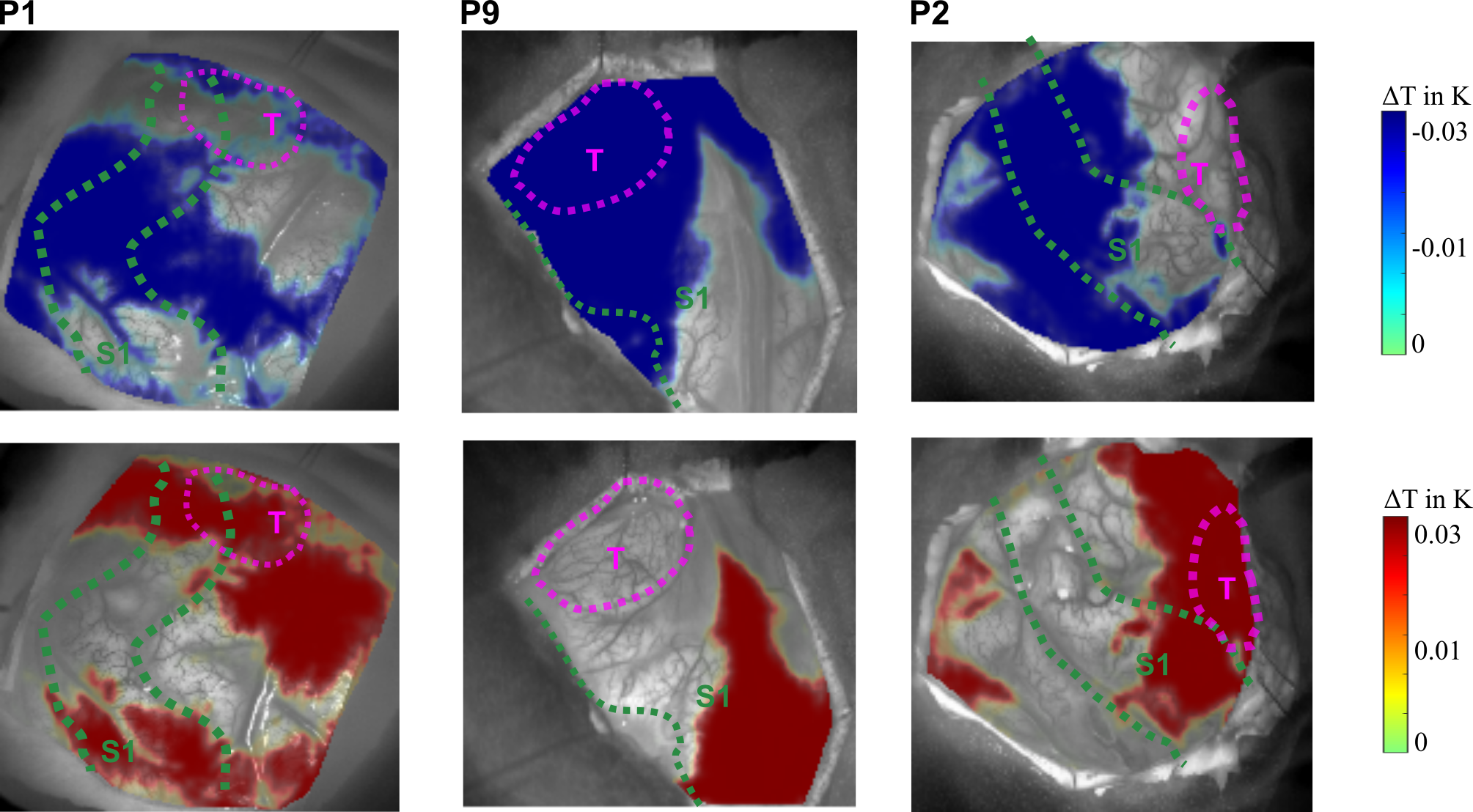


**Fig. Suppl. 2** Temperature dynamics during rest and stimulation phases for the three patients with cortical tumors. In the upper row regions where the temperature was lower during stimulation compared to rest are shown, indicating cooling during activation. Each colored pixel represents this cooling effect. In the lower row, areas with increased temperature during stimulation are highlighted, reflecting different behavior in tumor-affected regions (outlined in pink). These examples illustrate typical cooling patterns in S1 regions and distinct temperature increases in pathologically altered tissue.
